# Natural Products for the Prevention of Oxidative Stress-Related Diseases: Mechanisms and Strategies

**DOI:** 10.1155/2016/4628502

**Published:** 2016-01-26

**Authors:** Wei Chen, Zhenquan Jia, Min-Hsiung Pan, Pon Velayutham Anandh Babu

**Affiliations:** ^1^Department of Food Science and Nutrition, Zhejiang Key Laboratory for Agro-Food Processing, Zhejiang University, Hangzhou 310058, China; ^2^Department of Biology, The University of North Carolina at Greensboro, Greensboro, NC 27412, USA; ^3^Institute of Food Science and Technology, National Taiwan University, Taipei 10617, Taiwan; ^4^Division of Nutrition, College of Health, University of Utah, Salt Lake City, UT 84112, USA

Current opinions with respect to the etiology of chronic diseases such as neurodegenerative disease, cardiovascular diseases, and cancer are still controversial. There are multiple factors involved, and oxidative stress that results from the imbalance between reactive oxygen species and antioxidants is a one of the key factors in the development of these chronic diseases. Indeed, experimental and clinical evidences support the casual relationship between oxidative stress and various chronic diseases. Thus, numerous studies are focused on ameliorating these chronic diseases by reducing the oxidative stress. Epidemiological and clinical studies suggest that natural products can combat oxidative stress and reduce the morbidity and mortality associated with chronic diseases. Many natural compounds such as flavonoids are potential antioxidants that protect against reactive oxygen species (ROS) or reactive nitrogen species- (RNS-) induced damage and ameliorate oxidative stress-related diseases, such as neurodegenerative diseases, cardiovascular diseases, inflammatory conditions, and cancer. Although various natural products have been shown to possess potential protective effects against chronic diseases, the bioactivities of large number of natural compounds remain unknown. Therefore, understanding and validating the bioactivities of the natural compounds and the molecular mechanisms involved would provide solid scientific foundation to use the natural compounds for the prevention and treatment of oxidative stress-related diseases. The primary objective of this issue is to highlight the role of some of the natural products that ameliorate oxidative stress and reduce pathological complications associated with chronic diseases. The articles published in this special issue include four reviews and four original research articles that describe the importance of some of the natural products.

The review by J. Pérez-Hernández et al. is focused on the possible role of antioxidant herbal compounds as an alternative source for the treatment of neurodegenerative diseases (ND). The herbal compounds discussed in this review include polyphenols, flavonoids, alkaloids, and other miscellaneous antioxidant compounds from plants. An interesting review article by Y. Bai et al. discusses the cardiovascular beneficial effects of sulforaphane, a sulfur-based isothiocyanate compound in the cruciferous vegetables. Clinical and animal studies showed sulforaphane improved cardiovascular complications through activation of Nrf2. In this review, the authors discuss the possible molecular mechanisms involved in sulforaphane induced Nrf2 activation. J. A. Sirerol et al.'s review is focused on the role of natural compound stilbene in the prevention of cancer. The authors discuss about the studies that support the anticancer effects of stilbene, which also include few clinical trials. In this issue, the review article by J. Wang et al. discusses the latest advancements and up-to-date discoveries on the mechanisms of antioxidant activity of polysaccharides and glycoconjugates derived from natural products.

ROS play a major role in the aging process and significantly contribute to the aging related pathological complications. The antioxidant system is compromised with aging and the antioxidant levels are no longer sufficient to counteract the generation of free radicals. Exogenous antioxidants may be a promising strategy to suppress oxidative stress associated with aging. In this issue, the original research article by M. Nebbioso et al. shows that *α*-lipoic acid (ALA) and superoxide dismutase (SOD) can counteract senile neurodegenerative deterioration to the retina and optic nerve. This study indicates that the combination of ALA and SOD could reduce oxidative stress and thereby prevent nuclear degradation and the subsequent cell death.

In everyday life, humans are readily exposed to toxicants derived from various sources such as food (e.g., ethyl carbamate), pesticides (e.g., paraquat), and industry (e.g., tert-butyl hydroperoxide). These toxic substances trigger oxidative stress in the biological systems, which consequently lead to acute or chronic diseases. In this issue, W. Chen et al. reported that wild raspberry can improve the protective capacity against ethyl carbamate-induced oxidative damage in Caco-2 cells. Their results demonstrate that raspberry extract subjected to simulated gastrointestinal digestion could improve the cellular antioxidant activity and attenuate ethyl carbamate-induced oxidative damage in Caco-2 cells. A. J. Case et al.'s research article shows that* Aronia melanocarpa* concentrate attenuated paraquat-induced neurotoxicity. In this study,* Aronia* berry concentrate at low doses can restore the homeostatic redox environment of neurons treated with paraquat, while high doses exacerbate the imbalance leading to further cell death. This suggests that moderate levels of* Aronia* berry concentrate may prevent reactive oxygen species-mediated neurotoxicity whereas high doses may have toxic effects. The study by H. Lv et al. shows that Licochalcone A (Lico A) enhances Nrf2-mediated defense mechanisms against tert-butyl hydroperoxide (t-BHP) induced oxidative stress and cell death via Akt and ERK activation in RAW 264.7 cells. Further, their results indicate that Lico A might modulate HO-1 and scavenge ROS via the activation of the PI3K/Akt and ERK/Nrf2 signaling pathways.

In summary, this special issue covers a series of topics addressing the role of natural products as antioxidants counteracting oxidative stress-related chronic diseases. We hope that the articles brought in this special issue not only enrich our understanding of the therapeutic role of natural products on oxidative stress-related disease but also provide promising perspectives on future novel therapeutic agents development.

*Wei Chen* *Wei Chen* *Zhenquan Jia* *Zhenquan Jia* *Min-Hsiung Pan* *Min-Hsiung Pan* *Pon Velayutham Anandh Babu* *Pon Velayutham Anandh Babu*

